# Antiproliferative and proapoptotic effects of DODAC/synthetic phosphoethanolamine on hepatocellular carcinoma cells

**DOI:** 10.1186/s40360-018-0225-2

**Published:** 2018-07-11

**Authors:** Arthur Cássio de Lima Luna, Greice Kelle Viegas Saraiva, Gilberto Orivaldo Chierice, Henrique Hesse, Durvanei Augusto Maria

**Affiliations:** 1 Department of Biochemistry and Biophysics, Butantan Institute, 1500, Vital Brasil Avenue, Sao Paulo, 05503-900 Brazil; 2 Department of Medical Sciences, Medical School, University of Sao Paulo, Sao Paulo, Brazil; 3 Department of Biochemistry, Institute of Chemistry, University of Sao Paulo, Sao Paulo, Brazil; 4 Department of Chemistry and Molecular Physics, University of Sao Paulo, Sao Carlos, Brazil

**Keywords:** Liposomes, Nanomedicine, Hepatocellular carcinoma, Antitumoral alkylphospholipids

## Abstract

**Background:**

Current studies have demonstrated that DODAC/PHO-S (Dioctadecyldimethylammonium Chloride/Synthetic phosphoethanolamine) liposomes induces cytotoxicity in Hepa1c1c7 and B16F10 murine tumor cells, with a higher proportion than PHO-S. Therefore, our aim was to evaluate the potential of DODAC/PHO-S to elucidate the mechanism of cell death whereby the liposomes induces cytotoxicity in hepatocellular carcinoma Hepa1c1c7, compared to the PHO-S alone.

**Methods:**

Liposomes (DODAC/PHO-S) were prepared by ultrasonication. The cell cycle phases, protein expression and types of cell’s death on Hepa1c1c7 were analyzed by flow cytometry. The internalisation of liposomes, mitochondrial electrical potential and lysosomal stability were also evaluated by confocal laser scanning microscopy.

**Results:**

After treatment with liposomes (DODAC/PHO-S), we observed a significant increase in the population of Hepa1c1c7 cells experiencing cell cycle arrest in the S and G_2_/M phases, and this treatment was significantly more effective to promote cell death by apoptosis. There also was a decrease in the mitochondrial electrical potential; changes in the lysosomes; nuclear fragmentation and catastrophic changes in Hepa1c1c7 cells. The liposomes additionally promoted increases in the expression of DR4 receptor, caspases 3 and 8, cytochrome c, p53, p21, p27 and Bax. There was also a decrease in the expression of Bcl-2, cyclin D1, CD90 and CD44 proteins.

**Conclusion:**

The overall results showed that DODAC/PHO-S liposomes were more effective than PHO-S alone, in promoting cytotoxicity Hepa1c1c7 tumor cells, activating the intrinsic and extrinsic pathways of programmed cell death.

## Background

Despite great advances in the research and the development of new therapeutic strategies, cancer remains one of the leading causes of death worldwide. In 2014, there were an estimated 1,665,540 new cancer cases diagnosed and 585,720 deaths were expected in the United States of America in 2014, and it is expected to increase to over 24 million by 2035 [1, 2].

With regards to the limiting factors of the therapies currently available for the treatment of cancer, new treatments that are more effective and less harmful are necessary. Therefore, antineoplastic phospholipids (AFTs) and lipid precursors have emerged as a promising new classes of antitumor agents that do not target the DNA, however they change the plasma membrane turnover, inducing cell death, with a high selectivity for cancer cell [3, 4]. Edelfosine, miltefosine, perifosine, erucylphosphocholine and erufosine, represent this new class of AFTs, structurally related antitumor agents [5–7].

Synthetic phosphoethanolamine (PHO-S), an lipid precursor, amino-ethyl phosphoric ester, has been previously synthesized by our group [8–13]. We demonstrated that the treatment of B16F10 cells with PHO-S was able to inhibit cell proliferation and induce G_2/_M cell cycle arrest [13]. In another study, PHO-S caused anti-proliferative effects on HUVEC, by reducing cyclin D1 mRNA, VEGFR1 gene transcription and VEGFR1 receptor expression [10, 12].

In vitro studies demonstrated that PHO-S induced cytotoxicity and apoptosis via mitochondrial pathways, in leukemia cells. The results showed that PHO-S was able to provide antiproliferative effects on acute promyelocytic leukemia (APL) cell lines. PHO-S demonstrated its antiproliferative effect on APL cell lines, decreasing CD177+ and Gr-17+ in immature myeloid cells in bone marrow, spleen and liver [11]. Additionally, the PHO-S has exerted anti-tumor activities in several tumor cell lines, such B16F10 cells; Skmel-28 and Mewo cells (human melanoma); MCF-7 cells (human breast cancer) and ehrlich ascites tumor [8–10, 12, 13].

Recently, PHO-S was encapsulated in DODAC (Dioctadecyldimethylammonium Chloride) liposomes by our group and the liposomes were physico-chemically characterised [14, 15]. In vitro studies demonstrated the efficacy of DODAC/PHO-S liposomes in inducing cytotoxicity in B16F10 murine melanoma and Hepa1c1c7 murine hepatocellular carcinoma cells, with IC_50%_ values significantly lower than PHO-S treatment. It was observed that Hepa1c1c7 cells display greater sensitivity to the DODAC/PHO-S formulation when compared with B16F10 and HUVEC cells. However, the molecular mechanism responsible for the anti-tumor properties of DODAC/PHO-S has not been demonstrated [14, 15]. Consequently, our aim was to clarify the mechanism of cell death where DODAC/PHO-S liposomal formulation induces cytotoxicity in hepatocellular carcinoma Hepa1c1c17.

## Methods

### Liposomal formulation DODAC/PHO-S

Liposomal formulation DODAC/PHO-S were formulated (1:1) in water, in accordance with procedures previously published [8–10, 12, 13]. After sonication, the liposomes were sterilized by filtration.

### Cell culture

Hepa1c1c7 murine hepatocellular carcinoma (ATCC® CRL 2026) was cultured in αMEM medium (LGC Biotecnologia, Cotia, SP, Brazil) and supplemented with 10% fetal bovine serum in a humidified incubator at 37 °C and 5% CO_2_. The cell viability was verified using Trypan Blue exclusion test. Hepa1c1c7 was chosen for the study because of its easy reproduction in vivo studies.

### Cell cycle phases distribution

The Hepa1c1c7 cells at a cell density of 1 × 10^5^ cells/well (80 to 90% confluence) were treated with PHO-S (0.3–2.0 mM), DODAC/PHO-S 1:1 (0.3–2.0 mM), and empty DODAC (0.3–2.0 mM), for 12 h. After treatment, the cells were washed with PBS (phosphate buffered saline) and fixed by the addition of 3 ml of ice cold 70% ethanol. The cells cycle was evaluated after cell incubation with 1.8 μg/ml propidium iodide (PI) solution (Sigma-Aldrich, St. Louis, MO, EUA), for 30 min. in the dark. The analysis was performed using Biosciences FACSCalibur flow cytometer (Becton Dickinson, San Jose, CA, EUA) and 10,000 events were collected. Subsequently, the data was analysed by ModFit LT 3.2 software (Becton Dickinson, San Jose, CA, EUA).

### Determination of apoptotic and necrotic cells percentage by flow cytometric (Annexin-V/PI)

The Hepa1c1c7 cells were plated at 1 mL 1 × 10^5^ cells/well in 6 well plates. After 24 h, the cells were treated with PHO-S and DODAC/PHO-S, for 12 h. Then, the cells were washed three times with PBS at 4 °C and resuspended in 100 μL of PBS, following this, were incubated with 4 μg/mL of AnnexinV (Sigma Aldrich, EUA) and 1.8 μg/μL of PI (Sigma Aldrich, EUA), for 1 h at 37 °C.

Following incubation, the cells were centrifuged at 2000 rpm for 10 min at 4 °C and resuspended in 400 μL of binding buffer 1×, provided by the manufacturer. The analysis were performed using Biosciences FACSCalibur flow cytometer (Becton Dickinson, San Jose, CA, EUA) and 10,000 events were collected. Following this, the data was analysed using ModFit LT 3.2 software (Becton Dickinson, San Jose, CA, EUA).

### Expression markers analysis by flow cytometry

The analysis of protein expression were performed as previously published [15, 16]. In brief, the Hepa1c1c7 cells (1 × 10^5^ cells/well, the cell density reached 80 to 90% confluence), were treated with PHO-S (0.3–2.0 mM), DODAC/PHO-S 1:1 (0.3–2.0 mM), and empty DODAC (0.3–2.0 mM), for 12 h, were washed with PBS and resuspended in FACS buffer with 2.5% paraformaldehyde for 1 h. After washing, cells were again resuspended in a primary antibody specific for the proteins anti-CD44, anti-CD90, anti-p53, anti-p21, anti-p27, anti-Bax, anti-Bcl-2, anti-caspase-3, anti-caspase-8 (Abcam, Cambridge, MA, United States); anti- cytochrome c, anti-DR4 (Santa (Cruz Biotechnology Inc., Santa Cruz, EUA) and anti-cyclin D1 (Cell Signaling Technology, Danvers, MA), at a concentration of 1 μg/ml at 4 °C, for 1 min. The corresponding isotope antibody was used as a negative control and as a secondary antibody was used Goat anti Mouse IgG (H/L): FITC (AbD Serotec, Raleigh, NC, United States). The cells were pelleted, washed twice with PBS, then, fluorescence-activated cell sorting (FACS) analysis was performed on BD Biosciences FACs Calibur flow cytometer (Becton Dickinson, San Jose, CA, United States) using Cell Quest and Win MDI 2.9 softwares.

### Analysis of the internalisation of DODAC/PHO-S fluorescent liposomes by confocal laser scanning microscopy

DODAC/PHO-S liposomes 1:1 were prepared according to the protocol as described previously, adding 0.25 mol%, in relation to DODAC, of the fluorescent probe (Dil): 1,1′-dioctadecyl-3,3,3′,3′- tetramethylindocarbocyanine perchlorate (Life Technologies, CA, United States).

Aliquots of Hepa1c1c7 cells (1 × 10^5^ cells/well, the cell density reached 80 to 90% confluence), were plated on sterile glass slides. After adhesion, cells were treated with DODAC/PHO-S fluorescent liposomes (1:1) 0.3 and 2.0 mM. Then, cells were fixed by adding ProLong® and incubated at − 20 °C, in the dark. The analysis was realised by confocal laser scanning microscopy (Carl Zeiss LSM 700; Leica, Mannheim, Germany).

### Analysis of changes in mitochondrial electrical potential (ΔΨm) by confocal laser scanning microscopy

The analysis of the ΔΨm was realised by confocal laser. The Hepa1c1c7 cells were plated on sterile glass slides in 24-well plates, at concentration of 10^5^. After 24 h, the cells were treated with PHO-S and DODAC/PHO-S (1:1) at 0.3 and 2.0 mM, for 6 h. Then, the incubation with rhodamine-123 and analyses were perfomed as previously published [15, 16].

### Evaluation of lysosomal stability using acridine orange (AO)

The Hepa1c1c7 were plated on sterile glass slides in 24-well plates, at concentration of 1 × 10^5^ cells/well. After 24 h, the cells were treated with PHO-S and DODAC/PHO-S, for 6 h. The cells were then incubated with OA and analysed as previously published [15, 16].

### Statistical analysis

Statistical analyses were perfomed as previously detailed [15, 16]. In brief, the differences among measurements of the groups studied were analysed using One-way ANOVA (and non-parametric) and Kruskal Wallis test. *p*-values * < 0.05, ** < 0.01, and *** < 0.001 are statistically significant.

## Results

### DODAC/PHO-S liposomal formulation induces cell cycle arrest in G_2_/M

The compounds were evaluated for their ability to induce changes in the cell cycle. The Hepa1c1c7 cells were treated with PHO-S, DODAC/PHO-S (1:1) at a concentration of 0.3 and 2.0 mM, for 12 h. The exposure time was determined by taking into account the results of the cytotoxicity, as previously published, and performed simultaneously with this study [13, 14].

**Fig. 1:**
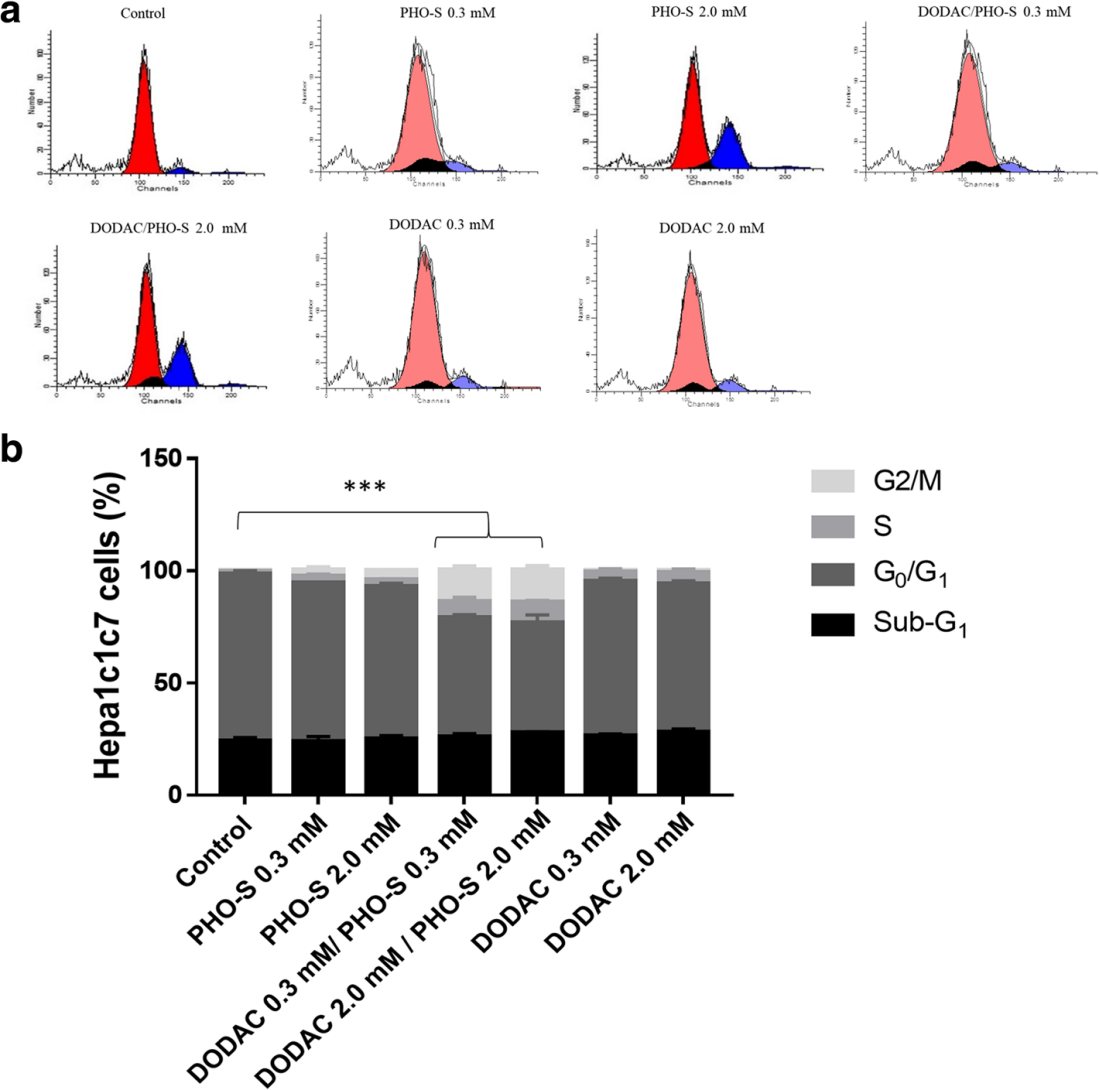
Analysis of cell cycle phases of Hepa1c1c7 cells treated with PHO-S, DODAC and DODAC/PHO-S. The cells were treated with different concentrations of the formulations for a period of 12 h. The graph shows the correlation of the effect expressed as a mean ± SD of three independent experiments. **a** Representative histograms of cell cycle analysis. Y-axis represent number of cells; X-axis represents DNA content (PI intensity). **b** Population of cells in sub-G1, G_0_/G_1_, S and G_2_/M  phase. Level of statistical significance * *P* < 0.05, ** *p* < 0.01 and *** *p* < 0.001

The distribution of Hepa1c1c7 cells, that were treated, suffered alterations in their percentage, compared to the control group (Fig. 1a and b). However, it is not possible to observe significant changes in the percentage of cells in the sub-G_1_ phase, among the treatments and control group (Fig. 1a and b). The number of cells in the G_0_/G_1_ phase decreased significantly with DODAC/PHO-S treatment, in relation to the control group (74.7 ± 1.1%), presenting a percentage of 53.2 ± 1.2% (0.3 mM) and 48.9 ± 3.5% (2.0 mM) (Fig. 1b).

The treatment with DODAC/PHO-S induced significant increases in the number of cells in S-phase, when compared to previous treatments, with values of 7.1 ± 1.9% (0.3 mM) and 9.1 ± 0.8% (2.0 mM) (Fig. 1b). The percentage of cells in the G_2_/M phase showed a significant increase when treated with DODAC/PHO-S liposomes, with values of 14.3 ± 1.7% (0.3 mM) and 14.3 ± 2.1% (2.0 mM) (Fig. 1b).

### DODAC/PHO-S induces an increase in the number of apoptotic cells

**Fig. 2:**
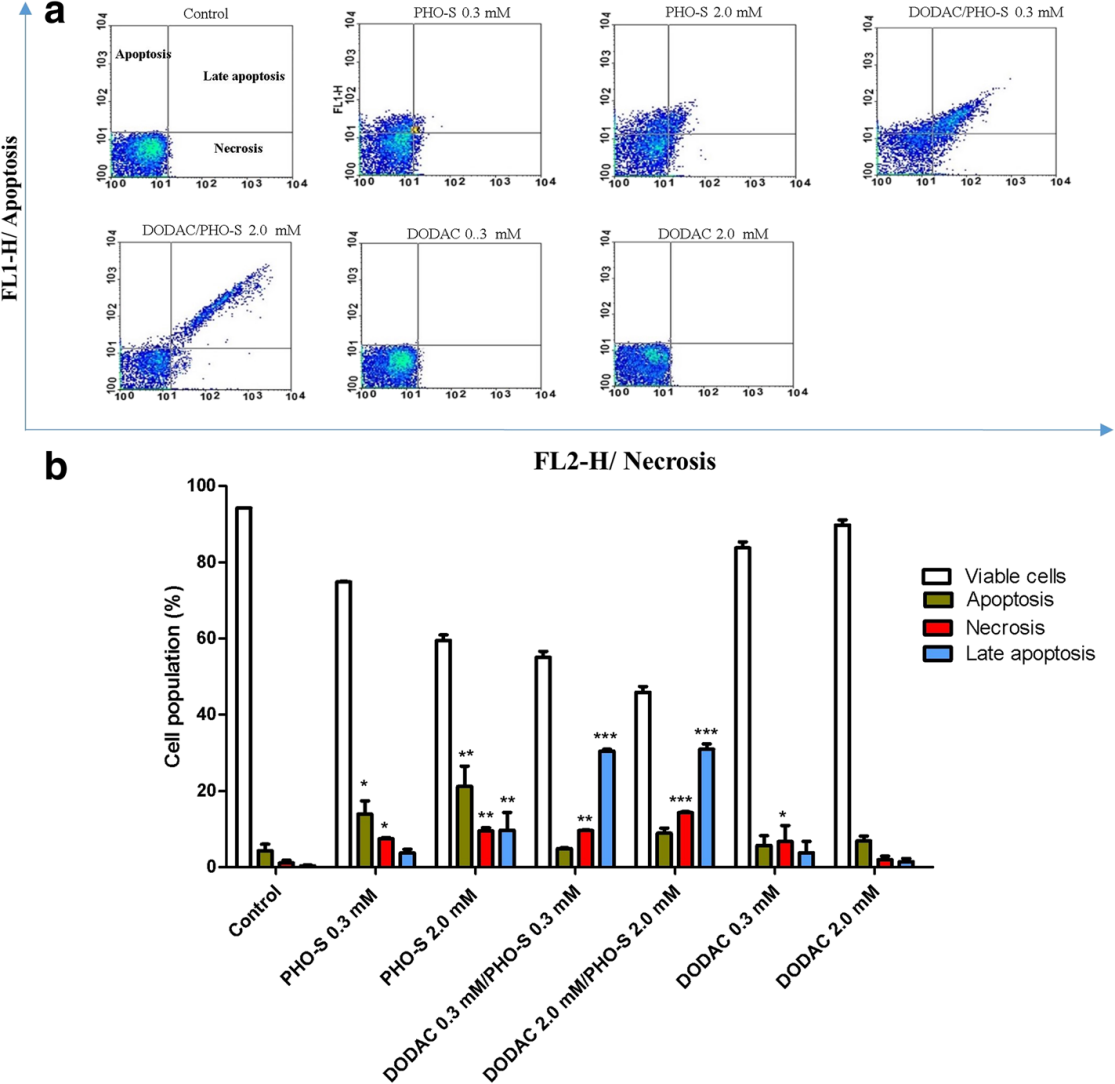
Evaluation of cell death mechanism by quantifying the percentage of Hepa1c1c7 cells undergoing apoptosis, late apoptosis and necrosis, by flow cytometry using Annexin V / PI kit. The Hepa1c1c7 cells were treated with different concentrations of PHO-S, DODAC/PHO-S and DODAC, for a period of 12 h. **a** representative dot plots of fluorescence levels obtained by staining the cells for Annexin V/PI kit. **b** The bar chart shows the correlation of the effect expressed as a mean ± SD of three independent experiments. Level of statistical significance * *P* < 0.05, ** *p* < 0.01 and *** *p* < 0.001

The Hepa1c1c7 cells were treated with PHO-S, DODAC/PHO-S (1:1) and DODAC (0.3–2.0 mM), for 12 h. Subsequently, cells in apoptosis, late apoptosis and necrosis were quantified by flow cytometry using FITC Annexin V/PI apoptosis detection Kit. The treatment with PHO-S (0.3 and 2.0 mM) significantly increased the number of apoptotic and necrotic cells, with values of 13.9 ± 3.4% (0.3 mM) and 7.5 ± 0.3% (0.3 mM), respectively. In the highest concentration (PHO-S 2.0 mM), the percentage of apoptotic cells was 21.2 ± 5.4% and 7.5 ± 0.3% of necrotic cells. Only in the highest concentration (2.0 mM) was it possible to observe an increase in the number of cells in late apoptosis, presenting a percentage value of 9.7 ± 4.6% (Fig. 2b).

The treatment with DODAC/PHO-S (0.3 and 2.0 mM) significantly increased the number of necrotic cells, with values of 9.6 ± 0.14% (0.3 mM) and 12.3 ± 0.3% (2.0 mM). There was also an increase in late apoptosis, with a percentage of 30.4 ± 0.4% (0.3 mM) and 31.0 ± 1.3% (2.0 mM). The treatment with DODAC (0.3 mM) increased the number of necrotic cells to a lesser extent when compared to the other treatments, with a percentage of 6.7 ± 0.9% (Fig. 2b).

### Internalisation of liposomes DODAC/PHO-S by Hep1c1c7 cells

**Fig. 3:**
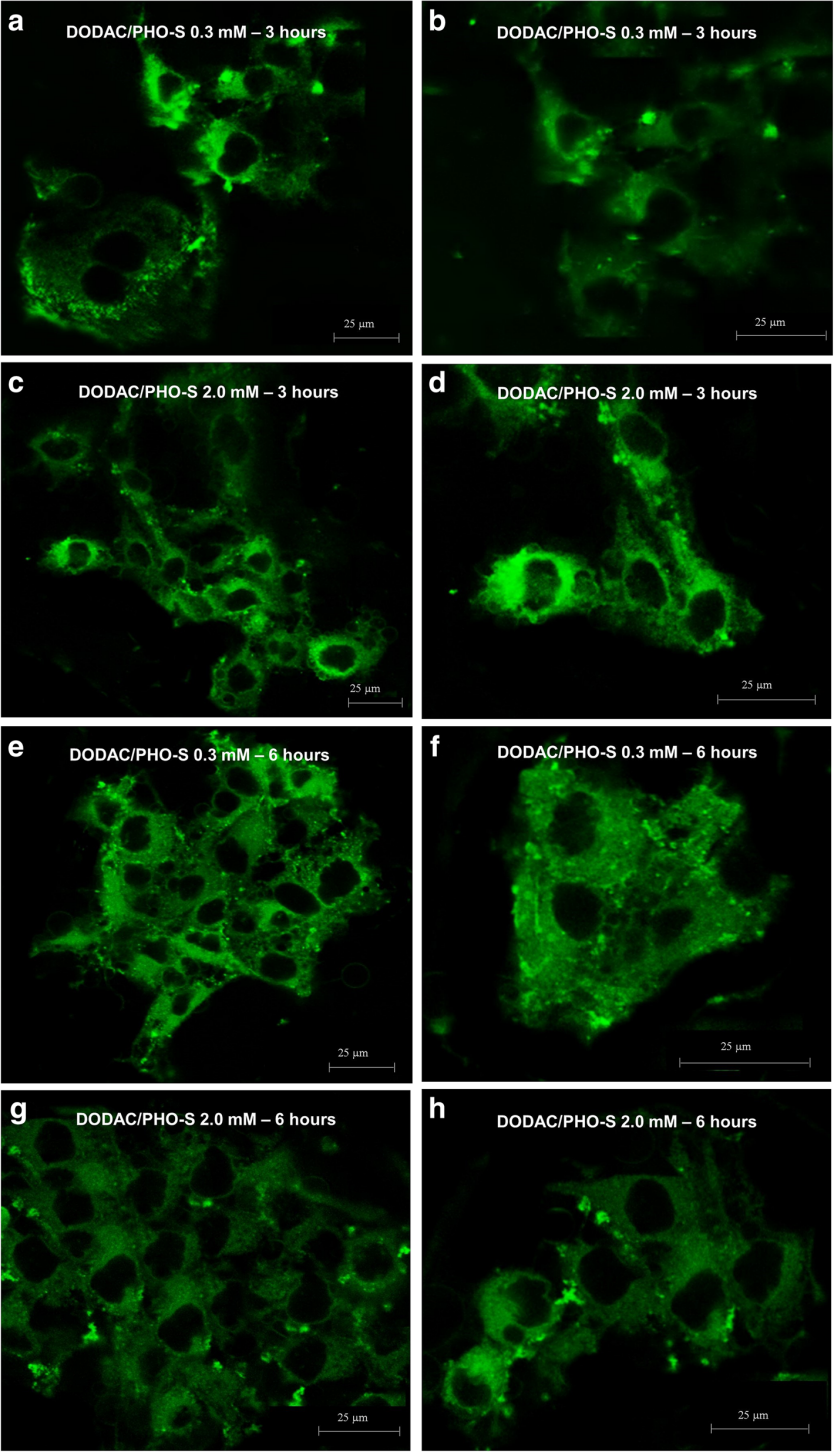
Photomicrographs of liposomes DODAC/PHO-S/Dil in Hepa1c1c7 cells analyzed by confocal laser microscopy. Cells were treated with the liposomal formulation (0.3 and 2.0 mM) with a fluorescent marker for a period of 3 to 6 h. Labeled liposomes can be observed and distinguished by the colour green in the cytoplasm of the cells. Cells treated with **a** and **b** DODAC/PHO-S 0.3 mM; **c** and **d** DODAC/PHO-S  2.0 mM, for a period of 3 h. Cells treated with **e** and **f** DODAC/PHO-S 0.3 mM; **g**, **e**, **h** DODAC/PHO-S 2.0 mM, for a period of 6 h

The internalisation of DODAC/PHO-S liposomes with fluorescent probe (Dil), was evaluated by confocal laser scanning microscopy. The Hepa1c1c7 cells were treated with DODAC/PHO-S liposomes (0.3 and 2.0 mM). The fluorescently labeled liposomes were observed and easily distinguishable by their green colour. After treatment, the presence of the DODAC/PHO-S fluorescently labeled liposomes (0.3 and 2.0 mM) were observed in the cytoplasm of the Hepa1c1c7 cells, confirming that internalisation of the liposomes occurred, in a short period of time (Fig. 3).

### DODAC/PHO-S liposomes decreases the ΔΨm

**Fig. 4:**
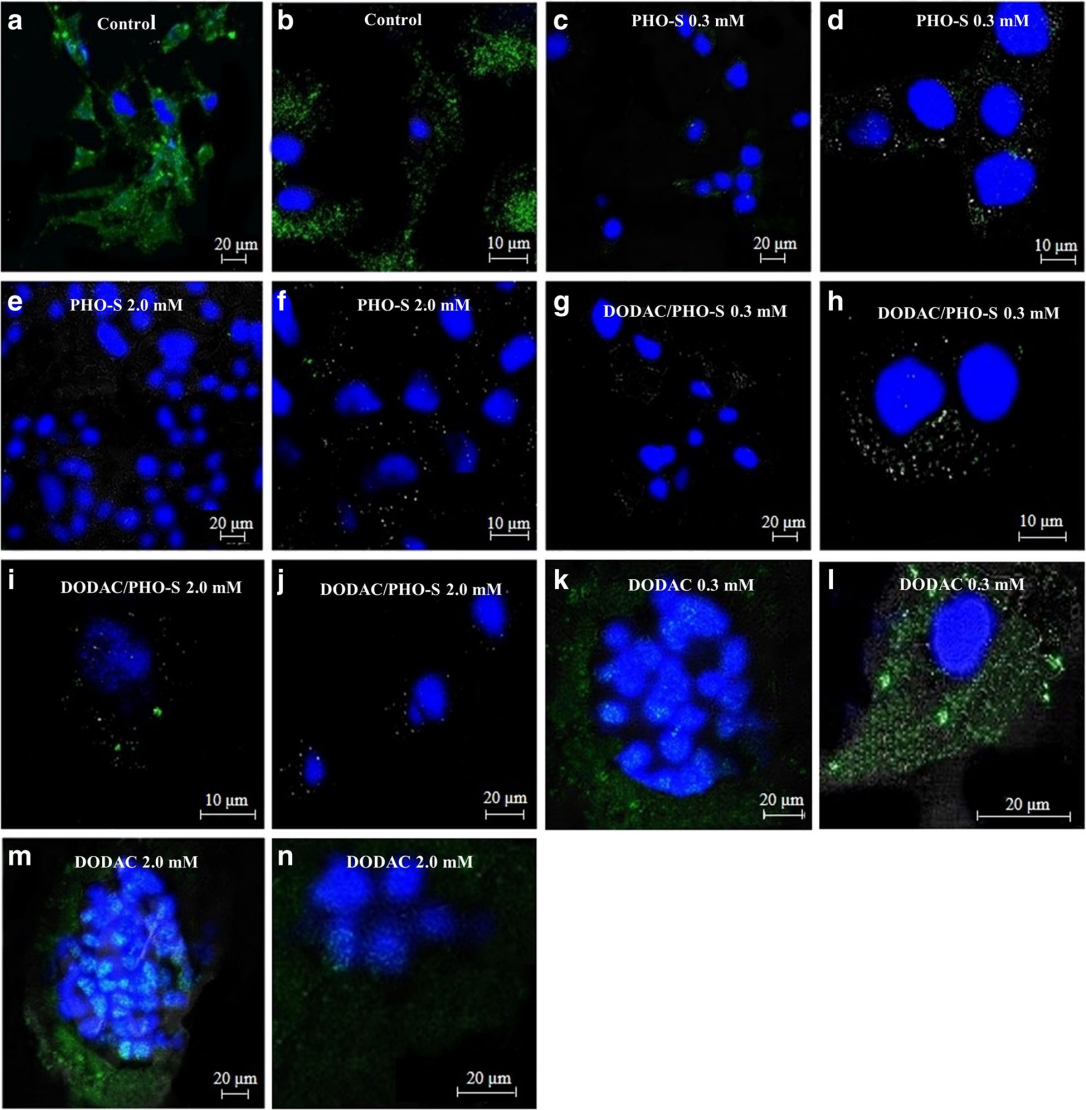
Photomicrographs of the Hepa1c1c7 cells mitochondria labeled with rhodamine 123 (green) and nucleus with DAPI (blue) and analyzed by confocal laser microscopy. Cells were treated with PHO-S, DODAC and DODAC/PHO-S 1: 1, for a period of 6 h and mitochondria were labeled with rhodamine 123. **a** and **b** control; **c** and **d** PHO-S -0.3 mM; **e** and **f** PHO-S 2.0 mM; **g** and **h** DODAC/PHO-S 0.3 mM; **i** and **j** DODAC/PHO-S 2.0 mM; **k** and **l** DODAC 0.3 mM; **m** and **n** DODAC 2.0 mM. Scale bar = 10 and 20 μm

The Hepa1c1c7 cells were treated with PHO-S, DODAC/PHO-S (1:1) and DODAC (0.3 and 2.0 mM), for a period of 6 h. The cells treated with PHO-S and DODAC/PHO-S, demonstrated a significant decrease in ΔΨm, when compared to the control group. The cells were treated with DODAC (0.3 and 2.0 mM) and presented an attenuation of fluorescent intensity, compared to the control group. However, the positive staining demonstrated that cells had functional mitochondria (Fig. 4).

### DODAC/PHO-S induces cellular morphological change

The Hepa1c1c7 cells were treated with PHO-S, DODAC and DODAC/PHO-S liposomes (2.0 mM), for a period of 6 h. The effects of these compounds in lysosomes were then evaluated with AO (acridine orange) assay, by confocal laser scanning microscopy. The nuclear DNA was labeled with DAPI (blue emission). For this assay, only the highest concentration (2.0 mM) was used, because it showed the greatest effectiveness in inducing cytotoxicity.

**Fig. 5:**
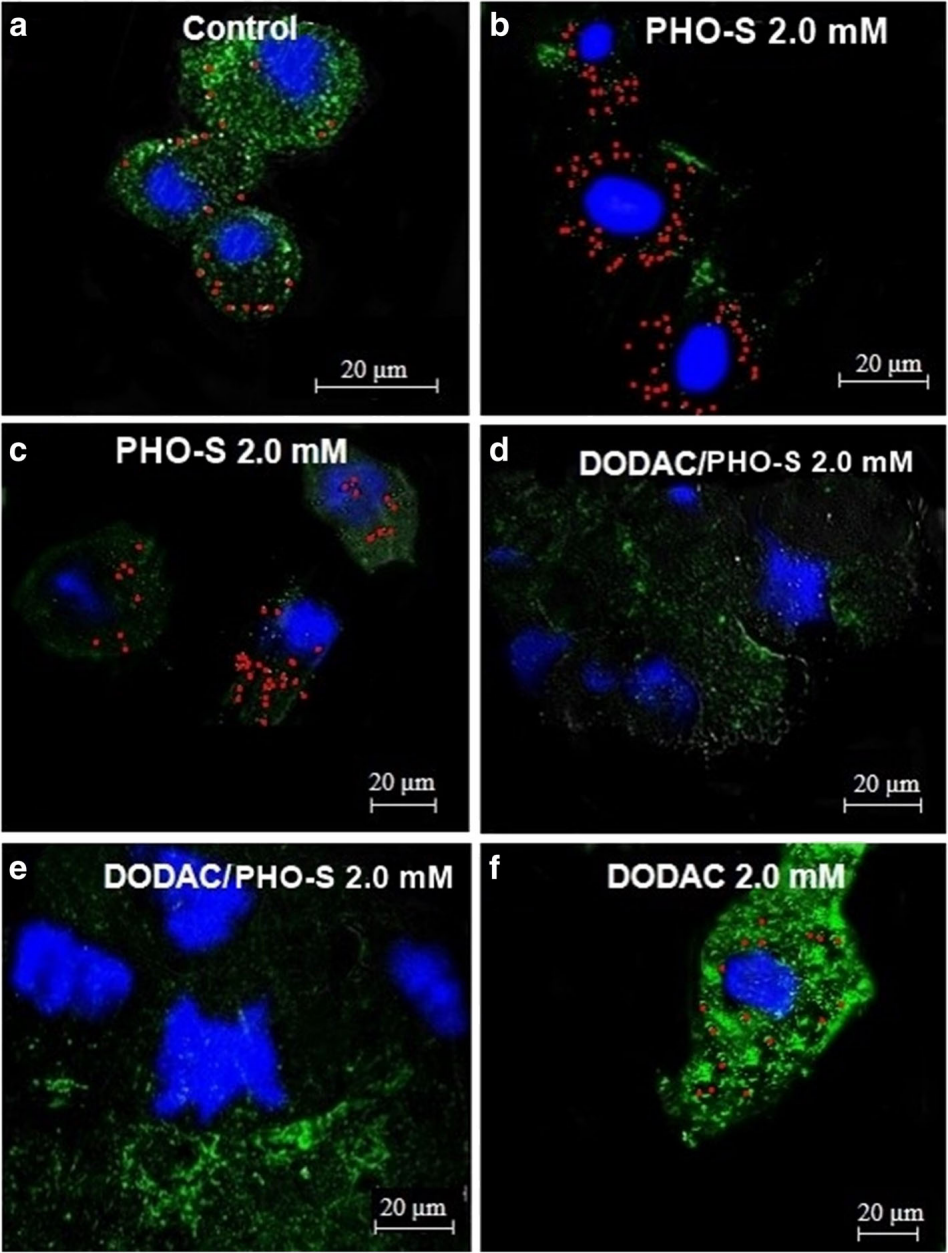
Photomicrographs of the cells of murine Hepa1c1c7 hepatocarcinoma lysosomes labeled with acridine orange (green) and nucleus with DAPI (blue) and analyzed by confocal laser microscopy. Cells were treated with PHO-S, DODAC and DODAC/PHO-S 1: 1, for a period of 6 h and lysosomes and nucleus were labeled. **a** control; **b** and **c**  PHO-S 2.0 mM; **d** and **e** DODAC/PHO-S 2.0 mM; **f** DODAC 2.0 mM. Scale bar = 20 μm

The control group cells demonstrated intense labeling of some lysosomes with a granular appearance, evenly distributed into cytoplasm (Fig. 5a). The same distribution pattern was observed in the cells treated with PHO-S. However, in this case the treatment induced an increase in the number of lysosomes (Fig. 5b and c). In the cells treated with DODAC/PHO-S liposomes, there decrease in the number of lysosomes and morphological alterations due to the apoptotic process were observed, as membrane bubbles and nuclear fragmentation (Fig. 5d and e). The cells treated with only DODAC presented a distribution pattern and labeling of lysosomes similar the cells of the control group (Fig. 5f).

### Protein expression involved in programmed cell death

Hepa1c1c7 tumor cells were treated with PHO-S (2.0 mM), DODAC and DODAC/PHO-S (0.3 and 2.0 mM), for 24 h. The expression of proteins involved in checkpoint, cell adhesion, regulation of the cell cycle and apoptotic pathway were quantified after the treatment. These proteins are as follows: TRAIL receptors 1 (DR4), active caspases-3 and -8, free cytochrome c, p53, p21, p27, CD44, CD90, Bax, Bcl-2 and cyclin D1.

**Fig. 6:**
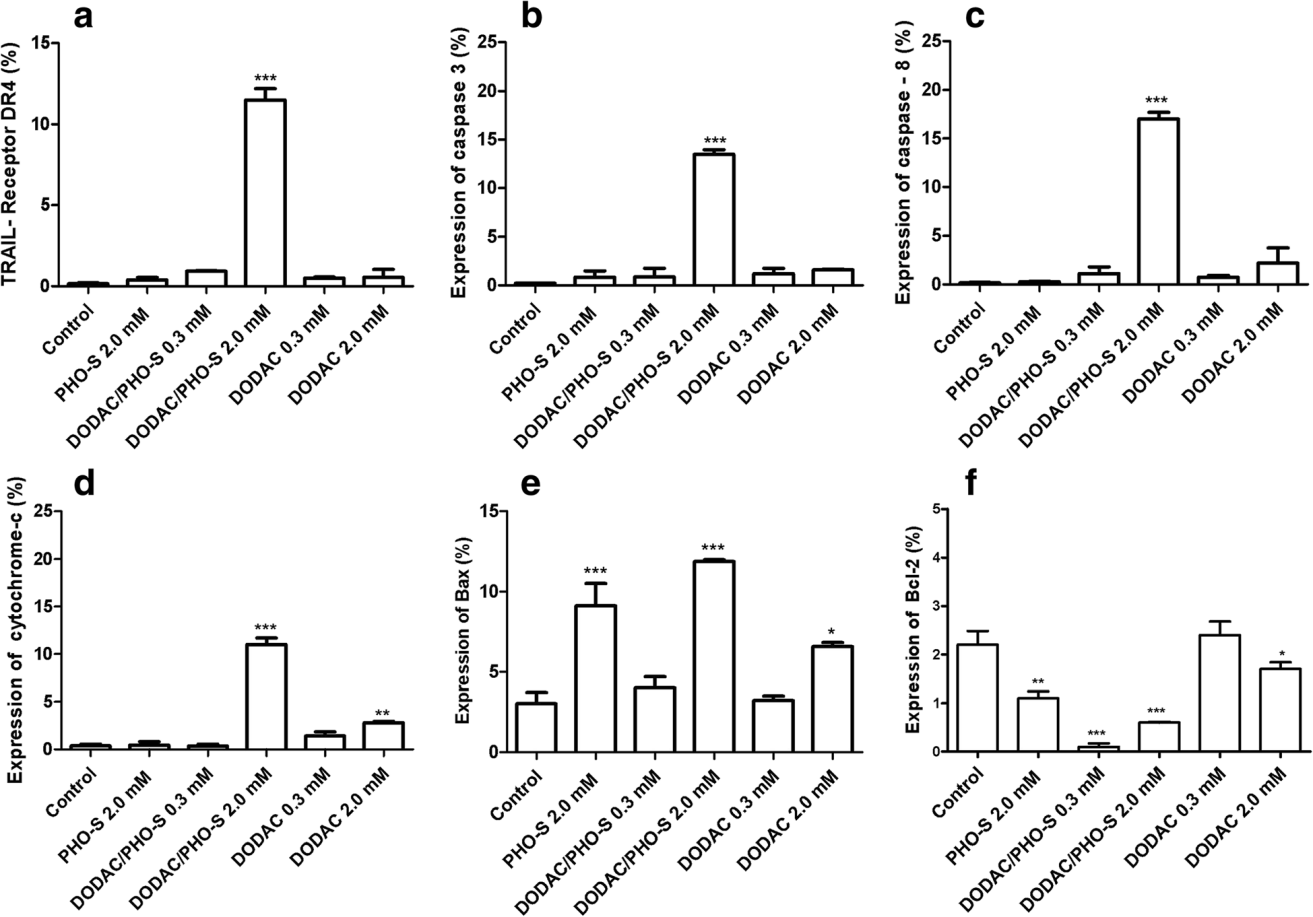
Analysis of protein expression in Hepa1c1c7 cells. The expression of proteins was quantified by flow cytometry after 24 h of treatment with PHO-S, DODAC and DODAC/PHO-S. The bar graphs show the level of expression of TRAIL - DR4 receptor (**a**); protein caspase 3 (**b**); caspase-8 protein (**c**); Free cytochrome c (**d**); protein Bax (**e**); protein Bcl-2 (**f**). Values are expressed as a mean ± SD standard deviation of three independent experiments. Level of significance * *P* < 0.05, ** *p* < 0.01 and *** *p* < 0.001

The Hepa1c1c7 cells treated with 2.0 mM DODAC/PHO-S, showed a significant increase in the expression of DR4 receptor, active caspases-3 and 8 and cytochrome c, with percentage values of 11.4 ± 0.1% (Fig. 6a), 13.5 ± 0.5% (Fig. 6b), 17.0 ± 0,7% (Fig. 6c) and 11.0 ± 0.7% (Fig. 6d), respectively. The treatment with empty DODAC (2.0 mM) also induced a significant growth in the expression of free cytochrome c, with value of 2.8 ± 0.1% (Fig. 6d).

Bax protein had an expression increase after treatment with 2.0 mM PHO-S (9.1 ± 1.2%), DODAC/PHO-S (11.9 ± 0.1%) and DODAC (6.5 ± 0.2%) (Fig. 6e). In contrast, antiapoptotic Bcl-2 protein had a decrease in the expression with 2.0 mM PHO-S (1.1 ± 0.9%), DODAC/PHO-S (0.3 mM - 0.1 ± 0.1%; 2.0 mM - 0.6 ± 0.1%) and 2.0 mM DODAC (1.7 ± 0.1%) (Fig. 6f).

**Fig. 7:**
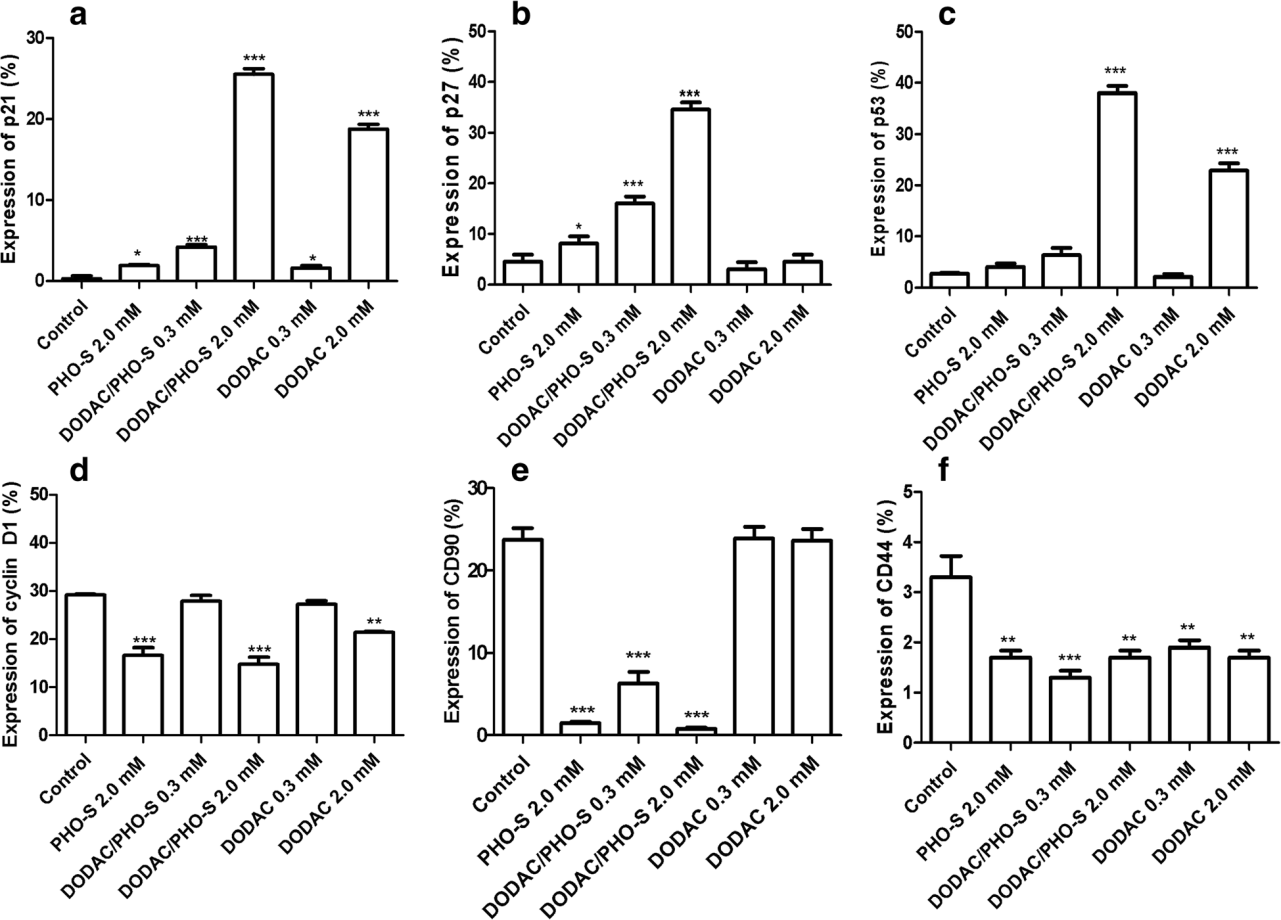
Analysis of expression of proteins involved in the proliferation and metastatic process in Hepa1c1c7 cells. The expression of the proteins was quantified by flow cytometry after 24 h of treatment with PHO-S, DODAC and DODAC/PHO-S. The bar graphs show the expression level of the protein p21 protein (**a**); p27 protein (**b**); p53 protein (**c**); cyclin D1 protein (**d**); CD90 marker (**e**) and CD44 marker (**f**). Values are expressed as a mean ± SD standard deviation of three independent experiments. Level of significance ** *p* < 0.01 and *** *p* < 0.001

The expression of p21 protein increased with 2.0 mM PHO-S (1.9 ± 0.1%); DODAC/PHO-S (0.3 mM - 4.3 ± 0.3% and 2.0 mM - 25.5 ± 0.7%) and empty DODAC (0.3 mM - 1.6 ± 0.3% and 2.0 mM - 18.8 ± 0.5%) (Fig. 7a). P27 protein had an increase in expression with 2.0 mM PHO-S (8.2 ± 1.6%); DODAC/PHO-S (0.3 mM - 16 ± 1.2% and 2.0 mM - 34.6 ± 0.9%) (Fig. 7b).

It was noticed that p53 protein increased with 2.0 mM DODAC/PHO-S (38 ± 1.7%) and 2.0 mM empty DODAC (22.9 ± 1.3%) (Fig. 7c). In contrast, the expression of cyclin D1 decreased with 2.0 mM PHO-S (16.7 ± 1.5%); 2.0 mM DODAC/PHO-S (14.8 ± 1.5%) and 2.0 mM empty DODAC (21.4 ± 0.1%) (Fig. 7d). Some markers as CD90 and CD44 were quantified after the treatment. It was possible to observe that CD90 decreased after treatment with 2.0 mM PHO-S (1.5 ± 0.9%) and DODAC/PHO-S (0.3 mM - 1.3 ± 0.1%; 2.0 mM - 1.7 ± 0.9%) (Fig. 7e). The expression of CD44 decreased significantly with all treatments (Fig. 7f). There were no significant differences among the treatments.

## Discussion

Several liposomal formulations containing antitumor agents are already clinically available or are under advanced stages of development, for example, Doxil® (Pegylated liposomal doxorubicin), Lipoplatin® (cisplatin) DepoCyte® (cytarabine), DaunoXome® (daunorubicin), MBP-426 (oxaliplatin), BIND (docetaxel) and MM30 (doxorubicin) [17–20]. Cationic liposomes or cationic polymers are often used in gene transfection due to their relatively easy complex formation with negatively charged genetic material [21, 22]. The complex polymer-DNA general mechanism is based on electrostatic interaction of the complex with DNA. This interaction allows internalisation of the DNA into the cells. The main advantages of these synthetic carriers are related to their versatility of their physicochemical properties, coupled with their easy handling and lower production cost [21–23].

In a recently published study, PHO-S was encapsulated in DODAC liposomes by our group [14, 15]. The physical, chemical stability and data relating to the characterisation of these liposomes have been mentioned before [14, 15]. This study demonstrated that PHO-S encapsulation was stable and showed an average of ∼50% of PHO-S [14, 15]. In vitro studies demonstrated that DODAC/PHO-S induced cytotoxicity and inhibited cell proliferation in B16F10 and Hepa1c1c7 cells. However this was not the case with normal cells with IC_50%_ values significantly lower than PHO-S treatment. It was also observed that the liposomes were able to induce their highest level of cytotoxicity in Hepa1c1c7 tumor cells [12, 13]. This could be due to the interaction of DODAC with lipid receptors of the tumor cells.

Recent studies have demonstrated that PHO-S inhibits cell proliferation and migration, leading to the accumulation of B16F10 and MCF-7 cells in the G_2_*/*M phase [12, 13]. As demonstrated in this study, the ability of DODAC/PHO-S liposomes to alter cell cycle phases in Hepa1c1c7 cells when compared to the control group. In the cells treated with these liposomes, there were significant increases in the population of Hepa1c1c7 cells experiencing cell cycle arrest at the S and G_2_/M phases. The treatment at the lowest and highest concentrations provided the same effect on the cell population, in these phases in the cycle. Therefore, demonstrating once again the sensitivity of Hepa1c1c7 cells to the treatment with DODAC/PHO-S liposomes, in relation to the effects observed in another study with B16F10 cells, performed simultaneously [14, 15]. The treatment with this liposomes, was able to maximize the cytotoxic effect of the treatment with PHO-S by mediating alterations in cell cycle. It was possible to distinguish cells that were arrested in these phases of the cell cycle by the increase in the proportion of cells in this period. However, as determined in the analysis of the ∆Ψm there was a greater number of unviable cells in the treated group, compared to the control group. This demonstrates that the cells are possibly activating the mechanism of programmed cell death.

The treatments performed only with empty DODAC liposomes resulted in a lesser increase in the number of Hepa1c1c7 cells in the S phase, however it did not increase the number of cells stopped in the G_2_/M phase, which is a specific targeted antitumor effect mediated by PHO-S [13]. This demonstrates the nonspecific effect of DODAC in promoting cytotoxicity, which is perhaps correlated with the ability of this compound to interact with electro-negatively charged molecules, including DNA [21, 22].

The induction of cell cycle arrest after DODAC/PHO-S treatment is very important, as one of the aims of the development of novel anticancer drugs is to formulate compounds that can act on cell cycle checkpoints, which are responsible for controlling cell cycle progression. The checkpoints occur at the entrance into the S phase (G_1_/S checkpoint), at the entrance into mitosis (G_2_/M checkpoint), as well as during replication (intra-S-phase checkpoint) [24]. A large proportion of conventional chemotherapy agents cause damage to DNA. In most cases, agents that cause DNA damage do not kill cancer cells directly, these agents act initially by activating the checkpoint and blocking cell cycle progression. This not only allows the cell to repair its DNA, but also to impede DNA synthesis or cell division in the presence of chromosome damage by initiating programmed cell death, via the intrinsic pathway [25]. To evaluate the mechanism of cell death mediated by liposomal formulation compared to PHO-S, the assay Annexin-V/PI was used. The evaluation of the integrity of the cellular membrane provides an indication whether the mechanism of cell death involves apoptosis or necrosis [26, 27]. Recent studies have shown the capacity of PHO-S in inducing death by apoptosis in different tumor cells [8–15, 28, 29]. The tests involving Annexin/PI verified these results and demonstrated that PHO-S is capable of stimulating apoptosis in greater proportion than necrosis. Substantiating the previous findings, the liposomal formulation of DODAC/PHO-S (0.3 and 2.0 mM) was significantly more effective when compared to PHO-S, in promoting cell death by apoptosis in Hepa1c1c7 cells and in increasing the proportion cells in late apoptosis. These results demonstrates the ability of the liposomal formulation to generate cytotoxicity more efficiently than the treatment with PHO-S.

The treatments with empty DODAC liposomes did not trigger cell death by apoptosis, with only a small increase in the number of cells killed by necrosis (with treatment at lower concentrations).

In Hepa1c1c7 cells, the liposomal formulation promoted cell death by inducing the activation of the intrinsic death pathway modulated by p53 activity. This protein is likely to have induced the activity of Bax and p21 proteins, and consequently the decreased expression of cyclin D1 protein, as shown in our results. Moreover, an increase of the pro-apoptotic p27 protein and decrease of the anti-apoptotic Blc-2 protein were observed. These results demonstrate the path or route that the DODAC/PHO-S liposomes possibly uses in order to induce cellular arrest in the G_2_/M phase. The expression of proteins involved in checkpoint, apoptosis and cell cycle regulation was greater in cells treated with the 2.0 mM DODAC/PHO-S than in the cells treated with PHO-S only. Thus indicating that treatment with the formulation is more effective than treatment with PHO-S.

There was no significant increase in p53 protein in the Hepa1c1c7 cells treated with PHO-S 2.0 mM. This effect was not observed probably by the exposure time of the cells to PHO-S because the expression of the proteins, which are regulated by transcriptional activation of p53, such as p21 and Bax, was changed. However, in the cells treated with the DODAC/PHO-S, the regulatory effect on p53 was sustained. This demonstrates the effectiveness of DODAC/PHO-S mediating mechanisms that cause cell cycle arrest and consequently die.

It is known that the mechanism through which p53 protein regulates the transition between the S and G_2_/M phase involves the regulation of Cdc2 kinase, which is essential for cells to enter mitosis [30]. Cdc2 is inhibited simultaneously by three transcriptional targets of p53: being Gadd45, p21, and 14–3-3. p53 is a key protein that has been shown to coordinate numerous cellular signaling pathways, as cell cycle and apoptosis. p53 induces the transcription of genes that negatively regulate the progression of cell cycle, as cyclin A, polo-like kinase 1 (PLK1), cyclin B1, cyclin B2, cyclin-dependent kinase 1 (CDK1), CDC20, cell cycle phosphatases CDC25A and CDC25C, DNA replication licensing factor MCM5, CKS116 and antiapoptotic survivin (BIRC5) [30–32]. Similarly, recent studies established that PHO-S not only promoted cell cycle arrest mediated by the action of p53, but also facilitated the decrease of the anti-apoptotic protein Bcl-2 in MCF-7 cells [12].

The significant increase in the expression of protein p27 in the treatment with the DODAC/PHO-S (2.0 mM) shows an important action mechanism of this formulation, because this protein acts as an inhibitor of kinases that are dependent on cyclins and thereby negatively regulates the progress of the cell cycle from G_1_ to S. In many neoplasms, the nuclear levels of p27 are reduced [33]. The downregulation of p27 occurs during the process of tumorigenesis, affecting proliferation and represents a negative response to the treatment. In normal cells, p27 blocks the progression of the cell cycle by inhibiting the activity of cyclin 2-CDK2 and cyclin A-CDK2 [33]. The induction of the expression of p27 is an important biomarker in response to chemotherapy, in this case for the liposomal formulation DODAC/PHO-S.

Considering the importance of cyclin D1 reduction in antitumor treatment, 2.0 mM liposomal formulation proved effective for this purpose because caused a decrease in cyclin expression in Hepa1c1c7 cells. Recent studies demonstrated the ability of PHO-S in reducing mRNA of cyclin D1 in MCF-7 cells and in reducing the expression of CDK4/6 in the model H460/Bcl-2 cells [12, 28]. The overexpression of cyclin D1 is associated with the development of resistance by the endocrine system in breast cancer cells. Therapeutic agents have been observed to induce the degradation of cyclin D1 in vitro, providing a useful approach for therapeutic intervention [34].

The checkpoint mechanism mediated by p53 protein may promote the repair of damaged DNA or lead to the activation of the mechanism of programed cell death. This is mediated by pro-apoptotic proteins such as Bax, PUMA and Noxa and decreased expression of anti-apoptotic proteins, including Blc-2. It was observed that treatment with DODAC/PHO-S liposomes promoted the activation of programed cell death mechanism, since there was an increase in the expression of Bax and decrease in the Bcl-2 proteins.

The treatments with empty DODAC liposomes promoted an increase in the expression of p53, p21 and Bax, and a decrease in Blc-2 proteins, though to a lower percentage that the treatment with the DODAC/PHO-S liposomes. DODAC can interact with the DNA and other negatively charged molecules, it is probably by inducing p53 protein activity and therefore the activity of p21, Bax and Blc-2. However, the use of this carrier was not cytotoxic to normal HUVEC endothelial cells, under the same proportions as compared to the B16F10 and Hepa1c1c7 tumor cell lines [15]. So, probably, indicates that it could likely, serve as an adjunct to anti-tumor effects exerted by PHO-S.

The induction of cell death by the intrinsic pathway in Hepa1c1c7 cells exerted by the DODAC/PHO-S (0.3 mM and 2.0 mM), was confirmed by the evaluation of ∆Ψm. The cells treated with the liposomal formulation decreased the ∆Ψm. Cells treated with only empty DODAC liposomes showed functional mitochondria. Recent study demonstrated that MCF-7 cells treated with PHO-S displayed disorders in their ∆Ψm [12].

After the treatment with liposomes DODAC/PHO-S the protein expression of extrinsic pathway was also evaluated, since PHO-S has been stated as a compound capable of forming a complex with DR4 and DR5 receptors. This represents a mechanism by which the AFTs are internalized into the cytoplasm by forming coaggregation with the TNF family receptors, in particular FasL [28, 35]. In non-small cell lung adenocarcinoma cells (H460WT), PHO-S probably uses the TRAIL pathway to induce apoptosis by activating the extrinsic pathway initiated by the activation of caspase 8. This mechanism is triggered when the signal for apoptosis is irreversible or insufficient. Thus, the mitochondrial pathway is recruited by the activation of caspase-3 mediated by the cleavage of Bid, a protein with a type BH3 domain [8, 9, 13]. The results obtained from this study showed that the DODAC/PHO-S increased the expression of the DR4 receptor and activated caspases 8 and 3, resulting in the release of cytochrome c, when compared to treatment with PHO-S. TRAIL family receptors (TNF-related apoptosis-inducing ligand), are often associated with death by apoptosis through the participation of DR4 and DR5 receptors [36].

The increased expression of pro-apoptotic proteins, as caspase 3 and 8, in the cells treated with DODAC/PHO-S, demonstrated that the encapsulation of PHO-S was able to potentialise its antitumor activity. Caspases are a family of cysteine proteases that serve as primary effectors during apoptosis, and have been categorised into two groups: initiator caspases (caspases 2, 8, 9 and 10) and effector caspases (caspases 3, 6 and 7). The cleavage of different substrates by caspases is mechanically responsible for the morphological and biochemical characteristics of apoptotic cell death [27, 37]. In this context, several studies have demonstrated that PHO-S increased the expression of caspase 3 and 8 and pro-apoptotic proteins [8–10, 12, 13]. Thus, in this work we demonstrated that liposome-encapsulated PHO-S was more efficient in promoting an increase in the pro-apoptotic proteins expression, as caspase 3 and cytochrome c and, consequently, trigger apoptosis.

Recent studies have shown that apoptosis triggered by TRAIL in hepatocellular carcinoma cells (Huh-7, Hep3B) was associated with the lysosomal permeabilization and consequently the increase of cathepsin B in the cytosol. The internalisation of TRAIL and its receptors can enable factors that induce permeabilization of the lysosomal membrane and culminate in the release of cathepsin B to the cytosol [38–40]. In this context, the obtained results suggest that the lysosomal pathway may be involved in the activation of cell death mediated by DODAC/PHO-S in Hepa1c1c7 cells, given that these liposomes promoted an increase in the expression of the DR4 receptor. During the validation of these results, it was observed in photomicrographs that Hepa1c1c7 cells treated with liposomes did not show any marks of intact lysosomes after 6 h of treatment, and there was also nuclear fragmentation and major changes in the cell cycle, as noted by the AO assay. Cells treated with PHO-S showed only an increase in the number of lysosomes, which demonstrates the effectiveness of the DODAC/PHO-S in inducing cytotoxicity. Cells treated only with empty liposomes DODAC also showed lysosomes with intact membranes. However, new studies should be conducted in order to confirm the participation of the lysosomal pathway as a mechanism of action of the liposomes DODAC/PHO-S.

The internalisation of the liposomes DODAC/PHO-S (0.3 and 2.0 mM), with only 3 h of treatment, was confirmed by confocal laser microscopy. It was observed that the liposomes are rapidly internalised, which makes it a great tool for antitumor therapy. After 6 h, it was still possible to observe liposomes in the cytosol of the cells. However, within this treatment time some cells already presented morphological changes corresponding to the apoptotic process.

The potential of liposomes DODAC/PHO-S in inducing cell death in Hepa1c1c7 cells, was confirmed by significant reduction in the expression of CD90 and CD44 markers. It demonstrated the ability of this formulation in the promotion of cytotoxicity in these cells. The cells treated with empty DODAC liposomes (0.3 and 2.0 mM) did not significantly reduce the expression of the marker CD90, therefore, these cells still display tumorigenic potential. Studies of patients with liver and in 91.6% of the blood samples taken CD45 and CD90^+^ were present. The CD44^+^/CD90^+^ cells showed a more aggressive phenotype than the CD90^+^/CD44^−^ cells and formed metastatic lesions in the lungs of immunodeficient mice. The blockade of CD44 expression avoided the local and metastatic formation of tumor nodules by CD90^+^ cells [41].

## Conclusion

The set of results demonstrate that electrostatic interaction between DODAC/PHO-S and cell membrane maximises the antitumor effects mediated by PHO-S. Thus, the overall results show that the liposomal formulation DODAC/PHO-S was effective in promoting cytotoxicity in Hepa1c1c7 cells, activating the intrinsic and extrinsic pathways of programmed cell death.

## Abbreviations

AFTs: Antineoplastic phospholipids
ANOVA: Analysis of variance
AO: Acridine Orange
APL: Acute promyelocytic leukemia
BH3: Bcl-2 homology 3
CD: Cluster of differentiation
Cyclin: Cell cycle protein
Dil: 1,1′-dioctadecyl-3,3,3′,3′- tetramethylindocarbocyanine perchlorate
DODAC: Dioctadecyldimethylammonium Chloride
FACS: Fluorescence-Activated Cell Sorting
h: hours
IgG: Immunoglobulin
Min: Minutes
PBS: Phosphate buffered saline
PHO-S: Synthetic phosphoethanolamine
PI: Propidium iodide
SD: mean ± standard deviation
TNF-α: Tumor necrosis factor alpha
αMEM: Minimum Essential Medium Eagle Alpha Modification
ΔΨm: mitochondrial electrical potential
